# Solitary Cutaneous Focal Mucinosis

**DOI:** 10.7759/cureus.18618

**Published:** 2021-10-09

**Authors:** Nikolas Gutierrez, Christof Erickson, Antoanella Calame, Philip R Cohen

**Affiliations:** 1 General Practice, 1st Marine Division, 1st Combat Engineer Battalion, Camp Pendleton, USA; 2 Dermatology, Compass Dermatopathology, San Diego, USA; 3 Dermatology, Scripps Memorial Hospital, La Jolla, USA; 4 Dermatology, University of California, Davis Medical Center, Sacramento, USA

**Keywords:** systemic disease, solitary, multiple, mucin, mucinosis, focal, cutaneous, colloidal iron

## Abstract

Solitary cutaneous focal mucinosis is a unique condition defined by the presence of mucin, a hyaluronic acid complex, in the dermis. The lesion typically presents as an isolated, asymptomatic papule or nodule on the extremities or back and is not associated with any systemic condition. Conversely, multiple cutaneous focal mucinosis present with numerous skin lesions has been found to be associated with systemic diseases such as scleromyxedema, systemic lupus erythematous, and thyroid disease. Therefore, additional laboratory investigation should be considered when multiple cutaneous focal mucinosis is discovered. The case of a 37-year-old man with solitary cutaneous focal mucinosis is discussed. The skin lesion presented as an asymptomatic nodule on his right upper shoulder; microscopic evaluation established the diagnosis, and laboratory investigation was negative for any associated conditions. Similar to previous reports of solitary cutaneous focal mucinosis, our patient provides additional supporting evidence that laboratory studies for mucin-associated systemic disease are not required for individuals who present with cutaneous focal mucinosis consisting of only a solitary skin lesion.

## Introduction

Mucinosis was first described as a solitary lesion by Johnson and Helwig in 1966 [2]. Cutaneous focal mucinosis is a skin condition characterized by the presence of mucin in the dermis. It can be primary (in which mucin is the main histologic finding resulting in a clinically distinct lesion) or secondary (which is associated with disorders in which mucin is an additional finding) [1-5].

Multiple cutaneous focal mucinosis can occur as an idiopathic condition; however, several of these individuals have a mucin-associated systemic disease. The associated systemic diseases include Birt-Hogg-Dube syndrome, scleroderma, scleromyxedema, systemic lupus erythematous, and thyroid disease. Therefore, it is reasonable to perform laboratory studies to assess mucin-associated systemic diseases in patients with multiple cutaneous focal mucinosis [1,5-10].

Solitary cutaneous focal mucinosis is considered a unique mucinosis that typically presents as an asymptomatic papule whose color can range from flesh-colored to white or red. The lesion usually appears on the extremities with the arms being more commonly affected than the legs. However, lesions on the head, neck, and trunk have also been described. In contrast to multiple cutaneous focal mucinosis, solitary lesions are not usually associated with systemic disease; therefore, laboratory studies are not required in these individuals [1-9].

The case of a 37-year-old man who presented with solitary cutaneous focal mucinosis is described. After biopsy established the diagnosis, he had an unremarkable laboratory investigation that confirmed that the single, asymptomatic benign lesion was not associated with any systemic disorders. The features of solitary cutaneous focal mucinosis and multiple cutaneous focal mucinosis are summarized.

## Case presentation

A healthy 37-year-old man with history of atypical nevi presented for his annual full body skin examination. He denied any new, enlarging, or irritating cutaneous lesions since his last evaluation. He was otherwise doing well without any concerns regarding his skin.

**Figure 1:**
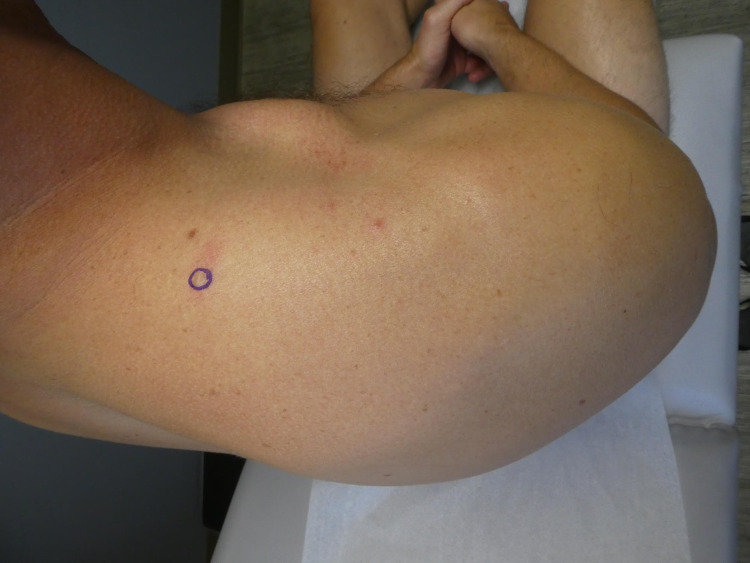
Clinical presentation of a solitary cutaneous focal mucinosis Posterior-superior view of the right upper shoulder of a 37-year-old man whose solitary cutaneous focal mucinosis presented as an asymptomatic isolated four-by-six millimeter skin-colored nodule (circled in purple).

On head-to-toe cutaneous examination, a four-by-six millimeter skin-colored nodule on his right upper shoulder was identified (Figure 1). Initial clinical impression included a differential diagnosis of a dermal adnexal cyst, a dermatofibroma, or a neurofibroma. The patient was amenable to a biopsy of the lesion in order to establish a definitive diagnosis.

**Figure 2:**
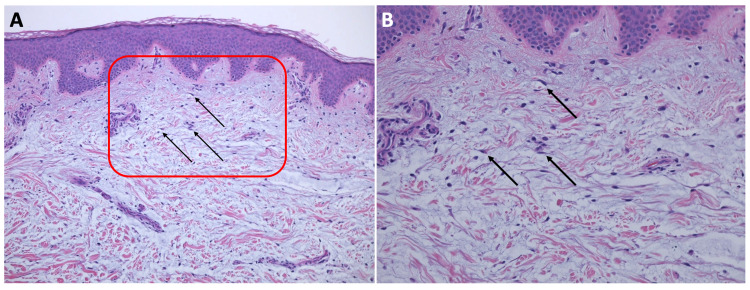
Microscopic presentation of hematoxylin and eosin-stained sections of a solitary cutaneous focal mucinosis on the right upper shoulder of a 37-year-old man (A) low and (B) higher magnification views of hematoxylin and eosin-stained sections of a solitary cutaneous focal mucinosis. The entire specimen contains the localized nodular basophilic-appearing deposition of mucin in the upper dermis. The stroma of the dermal connective tissue is loose and stellate fibroblasts are present (black arrows). The area enclosed within the red square in image (A) is shown at higher magnification in image (B). (Hematoxylin and eosin: (A): x10; (B): x20).

**Figure 3:**
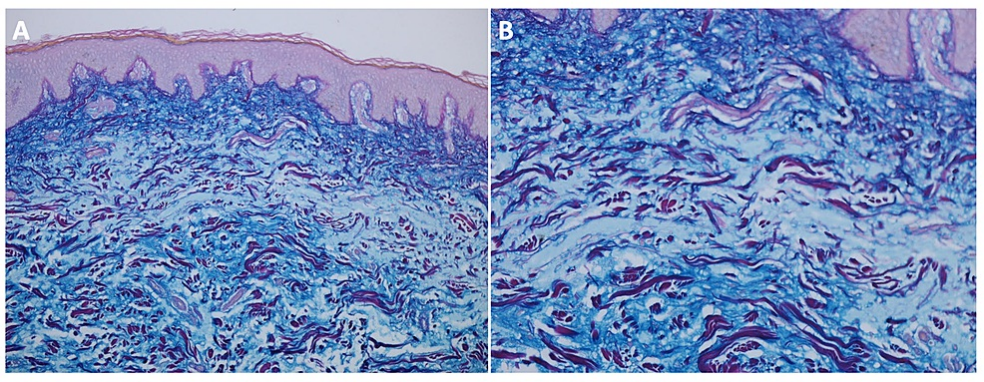
Microscopic presentation of colloidal iron-stained sections of a solitary cutaneous focal mucinosis on the right upper shoulder of a 37-year-old man (A) low and (B) higher magnification views of colloidal iron-stained sections of a solitary cutaneous focal mucinosis reveals positive deep blue staining that confirms that the composition of the nodular deposition that fills the upper dermis is mucin (Colloidal iron: (A): x10; (B): x20).

Microscopic evaluation of hematoxylin and eosin-stained sections revealed a nodular deposition of mucin associated with loose connective tissue stroma and stellate fibroblasts in the upper dermis (Figure 2). Colloidal iron-stained sections displayed dark blue staining of the nodular deposition, confirming the presence of mucin (Figure 3). Correlation of the clinical features and pathologic findings established the diagnosis of solitary cutaneous focal mucinosis.

The biopsy had removed most of the lesion, and no further treatment was required. After discussing the possibility of an incidental benign skin lesion versus the presenting cutaneous feature of a mucin-associated systemic disease, the patient wished to proceed with a laboratory investigation, which included antinuclear antibody (ANA), complete blood count (CBC), comprehensive metabolic panel (CMP), double-stranded deoxyribonucleic acid antibody (anti-dsDNA), hemoglobin A1c (HbA1c), ribonuclear protein antibody (anti-RNP), scleroderma antibody (scl-70), Sjogren syndrome antibody A (SS-A/Ro), Sjogren syndrome antibody B (SS-B/La), Smith antibody (Sm), thyroid stimulating hormone (TSH), triiodothyronine (T3), and thyroxine (T4). All of the laboratory studies were either negative or normal, ruling out an associated systemic condition.

## Discussion

Cutaneous focal mucinosis, first coined by Johnson and Helwig in 1966 [2], is a dermal degenerative condition characterized by increased mucin deposition in the dermis. Subsequently, Chen et al. in 2004 referred to the condition as a solitary soft fibroma-like polypoid mucinosis [5]. In 2016, Kuo et al. suggested that this lesion be called solitary cutaneous focal mucinosis to differentiate these patients from those who present with multiple lesions [7].

Solitary cutaneous focal mucinosis is a primary cutaneous mucinosis. The lesion has been reported in 182 individuals. Most of the patients were between the ages of 29 and 60, with a slight male predilection. The benign lesion typically presents as an asymptomatic dome-shaped papule or nodule on the extremities, more frequently observed on the arms than the legs. Its appearance ranges from flesh-colored to white to red. Given the variable and generic morphologic presentation, solitary cutaneous focal mucinosis is rarely clinically diagnosed; thus, a biopsy of the lesion is necessary to establish the diagnosis [1-3,7,9].

The predominant pathologic feature of cutaneous focal mucinosis is unencapsulated mucin, a hyaluronic acid complex, in the upper dermis. It can be visualized as light basophilic staining on hematoxylin and eosin-stained sections. Though diagnosis can be made on hematoxylin and eosin-stained sections, stains that demonstrate mucin (such as alcian blue, colloidal iron, and toluidine blue) can be helpful in confirming the diagnosis. The clinical and pathologic features of cutaneous focal mucinosis are summarized in Table 1 [1,2,4,7,8,10,11].

**Table 1:** Features of both solitary cutaneous focal mucinosis and multiple cutaneous focal mucinosis ANA: antinuclear antibody; anti-dsDNA: double-stranded deoxyribonucleic acid antibody; anti-RNP: ribonuclear protein antibody; CBC: complete blood count; CMP: comprehensive metabolic panel; HbA1c: hemoglobin A1c; Ref: references; scl-70: scleroderma antibody; Sm: Smith antibody; SS-A: Sjogren syndrome antibody A (Ro); SS-B: Sjogren syndrome antibody B (La); TSH: thyroid stimulating hormone; T3: triiodothyronine; T4: thyroxine ^a^Both solitary cutaneous focal mucinosis and multiple cutaneous focal mucinosis show the same histologic features. The overlying epidermis may be atrophic, hyperplastic, or normal. In the upper dermis, there is unencapsulated mucin, scattered fibroblasts, and diminished connective tissue elements such as collagen fibers, elastic fibers, and reticulum fibers. ^b^The color of the mucin after staining the tissue specimen is described.

Clinical feature	Solitary cutaneous focal mucinosis	Multiple cutaneous focal mucinosis	Ref
Clinical presentation	Single, asymptomatic nodule or papule whose color can range from flesh-colored to red to white	Multiple, asymptomatic nodules or papules whose color can range from flesh-colored to red to white	[2,7,8]
Location	Usually on upper back and upper extremities	No site predilection	[1]
Dermoscopy	Non-specific homogenous whitish pattern; a sharply demarcated yellow border was described in one patient	Non-specific homogenous whitish pattern	[11]
Histologic features^a^	Mucin and scattered fibroblasts in the upper dermis	Mucin and scattered fibroblasts in the upper dermis	[4]
Special stains^b^	Alcian blue: blue, Colloidal iron: deep blue, Toluidine blue: bluish-purple	Alcian blue: blue, Colloidal iron: deep blue, Toluidine blue: bluish-purple	[1,4]
Associated systemic disease	Not associated with systemic disease	Birt-Hogg-Dube syndrome, scleroderma, scleromyxedema, systemic lupus erythematous, and thyroid disease	[1,8,10]
Additional laboratory studies	None indicated	ANA, anti-dsDNA, anti-microsomal, anti-peroxidase, anti-RNP, anti-thyroglobulin, CBC, CMP, HbA1c, scl-70, Sm, SS-A, SS-B, TSH, T3, T4	[1]

The clinical differential diagnosis of solitary cutaneous focal mucinosis is extensive. In addition to dermal adnexal cyst, dermatofibroma, and neurofibroma that were suspected in our patient, other conditions to be considered in the differential diagnosis include basal cell carcinoma, epidermoid inclusion cyst, myxoma, nevus, and seborrheic keratosis. In nearly all cases, solitary cutaneous focal mucinosis was not suggested by the clinician who did the biopsy of the lesion [1-5].

The pathogenesis of cutaneous focal mucinosis is yet to be determined. Previous investigators have observed an association with trauma. Further research is required to elucidate the pathogenesis of this benign lesion [1].

A biopsy often provides adequate treatment of solitary cutaneous focal mucinosis. No further intervention is required if the lesion is completely removed. However, even after partial removal, solitary cutaneous focal mucinosis typically does not recur [1,6]. 

Previous reports suggest that solitary cutaneous focal mucinosis has not been associated with systemic disease; specifically, scleromyxedema, systemic lupus erythematous, and thyroid disease. In contrast, these conditions and other systemic diseases have been observed in patients who present with multiple cutaneous focal mucinosis. Therefore, although additional laboratory studies are not necessary for a solitary lesion of cutaneous mucinosis, a focused laboratory evaluation should be considered in patients with multiple cutaneous mucinosis lesions (Table 1) [1,2,4,7,8,10,11].

## Conclusions

Solitary focal cutaneous mucinosis is a unique benign primary mucinosis which presents as an isolated, asymptomatic papule or nodule that is not associated with systemic disease. The case of a 37-year-old man with solitary cutaneous focal mucinosis that presented as an asymptomatic nodule on his right upper shoulder is described; laboratory evaluation was negative for an associated disease. Our observation in this patient confirms those of previous researchers-laboratory investigation for a mucin-associated systemic disease in an individual with solitary cutaneous focal mucinosis is not required.
